# PLASMA PROTEOMIC MEDIATORS LINKING INTERMUSCULAR FAT WITH PSYCHOMOTOR SPEED

**DOI:** 10.1093/geroni/igae098.3689

**Published:** 2024-12-31

**Authors:** Tanaka Toshiko, Xianging Huang, Sadiya Khan, Iva Miljkovic, James Greg Terry, Teresa Tian, Caterina Rosano, Luigi Ferrucci

**Affiliations:** NIA/IRP/TGB/LSS, Baltimore, Maryland, United States; Northwestern University, Northwestern University, Illinois, United States; Northwestern University, Northwestern University, Illinois, United States; University of Pittsburgh, Pittsburgh, Pennsylvania, United States; Vanderbilt University Health Sciences, Nashville, Tennessee, United States; National Institute on Aging, Baltimore, Maryland, United States; University of Pittsburgh, Pittsburgh, Pennsylvania, United States; National Institute on Aging, Baltimore, Maryland, United States

## Abstract

Intermuscular fat (IMF), or muscular fat infiltration, has been inversely associated with measures of cognitive function. However, the mechanisms linking higher IMF and poor cognitive function are not fully understood. To explore this, we characterized plasma proteomic profile of IMF in Baltimore Longitudinal Study on Aging (BLSA, n=942), and validated the findings in the Coronary Artery Risk Development in Young Adults Study (CARDIA; n=2453) study. Plasma proteomics was measured using 7K SOMAscan, IMF was measured on CT scans of the thigh (BLSA) or abdomen (CARDIA), and processing speed was assessed with digit symbol substitution test (DSST). Associations between measures of IMF and proteins were assessed using linear regression adjusting for age, sex, education, self-reported race, and total muscle area. Of the 7268 plasma proteins tested, 718 proteins were significantly associated with IMF in both discovery and validation studies (FDR p<=0.05). Mediation analysis of the IMF -associated proteins, 28 proteins significantly mediated the inverse association between IMF and DSST. Pathway analyses show the biological process and molecular functions most enriched by these proteins were the synaptic structure and function (STLITRK3, CBLN2, NTRK3, EPHA4, C1QL3) pathway, and growth factor binding (HTRA1, IGFBP2, NTRK2, EGFR, IGFBP4, IGAFLS) pathways. These results suggest a unique proteomic signature of IMF that is consistent across two independent cohorts. Further, the association of higher IMF with lower cognitive processing speed may be at least partially mediated through dysregulation of synaptic functions and growth factors binding.

